# Diagnostic value of a new cryoprobe for peripheral pulmonary lesions: a prospective study

**DOI:** 10.1186/s12890-022-02003-0

**Published:** 2022-06-10

**Authors:** Midori Tanaka, Yuji Matsumoto, Tatsuya Imabayashi, Takuya Kawahara, Takaaki Tsuchida

**Affiliations:** 1 Department of Endoscopy, Respiratory Endoscopy Division, National Cancer Center Hospital, Tokyo, Japan; 2 Department of Thoracic Oncology, National Cancer Center Hospital, Tokyo, Japan; 3 Clinical Research Promotion Center, The University of Tokyo Hospital, Tokyo, Japan

**Keywords:** Cryobiopsy, Peripheral pulmonary lesion, Radial endobronchial ultrasound, Transbronchial biopsy, Lung cancer

## Abstract

**Background:**

Cryobiopsy is an established technique that yields larger and higher-quality samples than does a forceps biopsy. However, it remains underutilised in the diagnosis of peripheral pulmonary lesions (PPLs), mainly because of difficulties in handling conventional cryoprobes. A recently introduced single-use cryoprobe with a smaller diameter and more flexibility than conventional ones may improve its diagnostic ability for PPLs. We conducted this prospective study to evaluate the feasibility of transbronchial cryobiopsy in the diagnoses of PPLs, using a new 1.7-mm cryoprobe.

**Methods:**

The study included patients with PPLs less than 30 mm in diameter scheduled to undergo bronchoscopy. All the procedures were performed using a combination of virtual bronchoscopic navigation, radial endobronchial ultrasound (R-EBUS) and X-ray fluoroscopy, and all the samples were collected using the cryoprobe alone. Thereafter, we assessed the diagnostic outcomes and safety profiles.

**Results:**

A total of 50 patients were enrolled and underwent cryobiopsy. The median lesion size was 20.8 mm (range, 8.2–29.6 mm), and the negative bronchus sign was seen in 34% of lesions. The diagnostic yield was 94% (95% confidence interval, 83.5–98.8%). A positive bronchus sign had a significantly higher diagnostic yield than did a negative bronchus sign (100% vs. 82.4%; *P* = 0.035). The yield was achieved regardless of other variables, including lesion size, location, and R-EBUS findings. The major complications were mild and moderate bleeding in 28% and 62% of patients, respectively. Pneumothorax was identified in one patient.

**Conclusion:**

Transbronchial cryobiopsy using the new 1.7-mm cryoprobe is a feasible procedure that has the potential to increase the diagnostic accuracy for PPLs.

***Trial registration*:**

Japan Registry of Clinical Trials, jRCT1032200065. Registered July 8 2020, https://jrct.niph.go.jp/en-latest-detail/jRCT1032200065

## Background

Transbronchial biopsy is a widely used technique for diagnosing peripheral pulmonary lesions (PPLs) [1]. The introduction of advanced guidance modalities, such as radial endobronchial ultrasound (R-EBUS) [2–5], electromagnetic navigation bronchoscopy [6], and virtual bronchoscopic navigation (VBN) [7–10], has improved the diagnostic yield of bronchoscopy for PPLs. However, its diagnostic sensitivity remains insufficient (approximately 70%) regardless of the guidance techniques used [11]. This could be explained by the limitations of the sampling techniques. Currently available sampling devices for bronchoscopy, including forceps, needles, and brushes, have remained unchanged over the last few decades [1]. Although forceps have been the most commonly used tools in biopsies, the major limitations of such biopsies are small tissue sample sizes and the presence of crush artifacts, which can make the evaluation of histological findings difficult [12].

In recent years, transbronchial cryobiopsy has been introduced as a new sampling technique [12]. Compared to forceps biopsy, cryobiopsy can yield larger specimens with fewer artifacts and better architectural preservation, thereby allowing pathologists to easily assess histological findings [12–15]. Improvements in the quantity and quality of tissue samples may help increase the diagnostic yield of bronchoscopy. In fact, cryobiopsy has already been established to be superior to forceps biopsy for diagnosing interstitial lung diseases and endobronchial tumours [14–17].However, cryoprobes have not yet been commonly used to diagnose PPLs, mainly because of the difficulties in handling them [18–23]. The major limitations of the current cryoprobes with diameters of 1.9 or 2.4 mm in approaching PPLs are their thickness and stiffness. Thus, the diagnostic accuracy has not been acceptable as the accurate insertion of the cryoprobe to some angulated bronchi, such as the upper lobar bronchus, requires advanced techniques [21, 23]. Moreover, their reusable design may lead to an inevitable decrease in cooling performance due to mechanical stress and reprocessing through repeated use [24].

Recently, new single-use cryoprobes with smaller diameters have been developed [25, 26]. The new 1.1- and 1.7-mm cryoprobes are smaller and more flexible than the conventional reusable probes that have diameters of 1.9 mm. These new cryoprobes are expected to improve access to PPLs and sampling capability, thereby overcoming the limitations of conventional cryoprobes and forceps biopsies. An evaluation of the new cryoprobes in an in vivo animal model revealed that they were feasible and provided larger and higher-quality specimens than did forceps biopsy [24, 25]. Therefore, we conducted this prospective study to investigate the feasibility of the new 1.7-mm cryoprobe in diagnostic bronchoscopy for PPLs.

## Methods

### Study design and population

This was a prospective, single-centre observational study. The study protocol was approved by the National Cancer Center Institutional Review Board (No.2020–116), and the study was registered at the Japan Registry of Clinical Trials (jRCT1032200065). All included patients signed informed consent forms prior to inclusion in the study.

Since July 2020, consecutive patients were prospectively enrolled. Patients with a solitary pulmonary nodule suspected of being malignant were referred to our division for diagnostic bronchoscopy, and were considered for inclusion in this study. The inclusion criteria were as follows: age over 20 years, a lesion with a diameter less than 30 mm detected using computed tomography (CT), lesion located beyond subsegmental bronchi, absence of any treatment history for the lesion (i.e., surgery, radiation therapy, or anti-tumour drugs), and the possibility of discontinuing anticoagulant and antiplatelet drugs prior to bronchoscopy if the patients had been receiving them. The exclusion criteria were as follows: a treatment history of the lung or bronchi on the same side as the target lesion, presence of a tracheostomy hole, uncontrollable mental disorders or dementia, or the presence of any factor that precluded inclusion in the study as determined by the investigators.

All patients underwent high-resolution CT (HRCT) and chest plain radiography within 1 month before the bronchoscopy to evaluate the size, lobe, location, morphology, distance from the costal pleura, bronchus sign, related bronchial generation, and visibility of the lesion on a plain radiograph. The size was determined as the largest diameter on axial HRCT images. The location was defined as “inner” if the lesion was in the central two-third area from the pulmonary hilum and “outer” if the lesion was in the remaining peripheral one-third. The bronchus sign was defined as positive when the related bronchus directly led into the lesion.

### Procedure

All the procedures were performed by three bronchoscopists (M.T., Y.M., and T.I.), under moderate to deep sedation using local anaesthesia. A thin bronchoscope (BF-P260F or BF-P290; Olympus, Tokyo, Japan) was introduced through the oral route, and an endotracheal tube (Portex® Uncuffed Ivory PVC, Oral/Nasal Tracheal Tube 8.0-mm, Smiths Medical, Minneapolis, USA) was inserted. First, the target bronchus was assessed endoscopically, and if the target lesion was visible, the patient was excluded from the analysis. Thereafter, an R-EBUS probe (UM-S20-17S, Olympus) was inserted into the related bronchus through the working channel and advanced toward the target lesion. The position of the R-EBUS probe was adjusted under X-ray fluoroscopic guidance (VersiFlex VISTA®, Hitachi Ltd, Tokyo, Japan) referring to VBN and virtual fluoroscopy. These virtual images were constructed from high-resolution computed tomographic images using a workstation (Ziostation2®, Ziosoft, Tokyo, Japan) before the procedure [9, 27]. We divided the R-EBUS findings into three groups, “within,” “adjacent to,” and “invisible,” according to the positional relationship between the probe and the target [3]. If the lesion could not be detected using R-EBUS, the lesion was considered invisible and excluded from the analysis.

The R-EBUS probe was withdrawn after confirming the location of the targeted lesion. Subsequently, a 1.7-mm cryoprobe connected to ERBECRYO® 2 (Erbe, Tuebingen, Germany) was inserted through the working channel. After freezing for 3 s, the cryoprobe was removed together with the bronchoscope. Immediately thereafter, another therapeutic bronchoscope (BF-1T260 or BF-1TQ290, Olympus) was advanced through the tube and wedged into the biopsy site to control bleeding. The frozen specimens were thawed in saline and transferred to 10% neutral buffered formalin for histological examination. The remaining saline was used as the fluid specimen for cytological and microbiological examinations. Additionally, multiple aspects of the first tissue sample were stamped onto two glass slides. One slide was air-dried and stained using the Diff-Quik stain for rapid on-site cytological evaluation, and the other was fixed with ethanol and subjected to Papanicolaou staining. The entire procedure was repeated several times until a maximum of four specimens were obtained under X-ray fluoroscopic guidance, together with R-EBUS confirmation when needed, if there were no complications that contraindicated additional biopsies. For each biopsy, the number of attempts to obtain a sample was recorded.

### Endpoints

The primary endpoint was the diagnostic yield, which was defined as the percentage of patients whose definite diagnosis via cryobiopsy matched the final diagnosis. A definite diagnosis via cryobiopsy implied either malignancy, including cytological class IV/V, or specific benign results (e.g., granuloma, fibrosis, or inflammation based on histopathological/microbiological evidence). If the result was non-specific (i.e., peripheral lung and/or peribronchial tissue), it was considered nondiagnostic. The final diagnosis was confirmed by comprehensive analysis of evidence, including histological analysis after surgery or additional procedures, such as transthoracic needle biopsy, and treatment after the bronchoscopy. If none of the above applied, the final diagnosis was considered unknown.

The secondary endpoints were as follows: safety, sensitivity, specificity, accuracy, diagnostic yield by cytologic specimen, diagnostic possibility when using each tissue sample, and cumulative yield.

### Safety assessment

All adverse events occurring during and up to 2 weeks after the procedure were recorded. Among the possible complications, bleeding was categorised according to the following classification: mild, requiring less than 1 min of suctioning or wedging of the bronchoscope; moderate, suctioning more than 1 min or need for rewedging of the bronchoscope or instillation of cold saline, vasoactive substances, or thrombin; severe, selective intubation with an endotracheal tube for less than 20 min; and life-threatening, selective intubation over 20 min, admission to the intensive care unit, transfusion, or resuscitation, as described in the standardised definitions of bleeding [28].

### Statistics

We determined the sample size to confirm that the diagnostic yield of cryobiopsy exceeded 70%, i.e., the current diagnostic yield of transbronchial biopsy for PPLs [11]. When we hypothesised the diagnostic yield was approximately 85%, a sample size of 46 produced a two-tailed exact 95% confidence interval (CI) for a diagnostic yield with a lower limit of 70.2% and upper limit of 93.1%. Therefore, we planned to recruit a total of 50 patients. Continuous data were described as medians (ranges), whereas categorical data were presented as frequencies and percentages. The diagnostic yield was the ratio of correctly diagnosed cases using cryobiopsy specimens to the total number of patients expressed as a percentage. An exact 95% CI for the diagnostic yield was calculated. Comparisons of the diagnostic yields between factors considered to affect the diagnostic yield were evaluated using the chi-square or Fisher’s exact test when appropriate. A two-tailed *p*-value of less than 0.05 was considered statistically significant. Statistical analysis was performed by T.K. using Base SAS and SAS/STAT version 9.4 software of the SAS System for Windows (SAS Institute, Cary, USA).

## Results

**Fig. 1:**
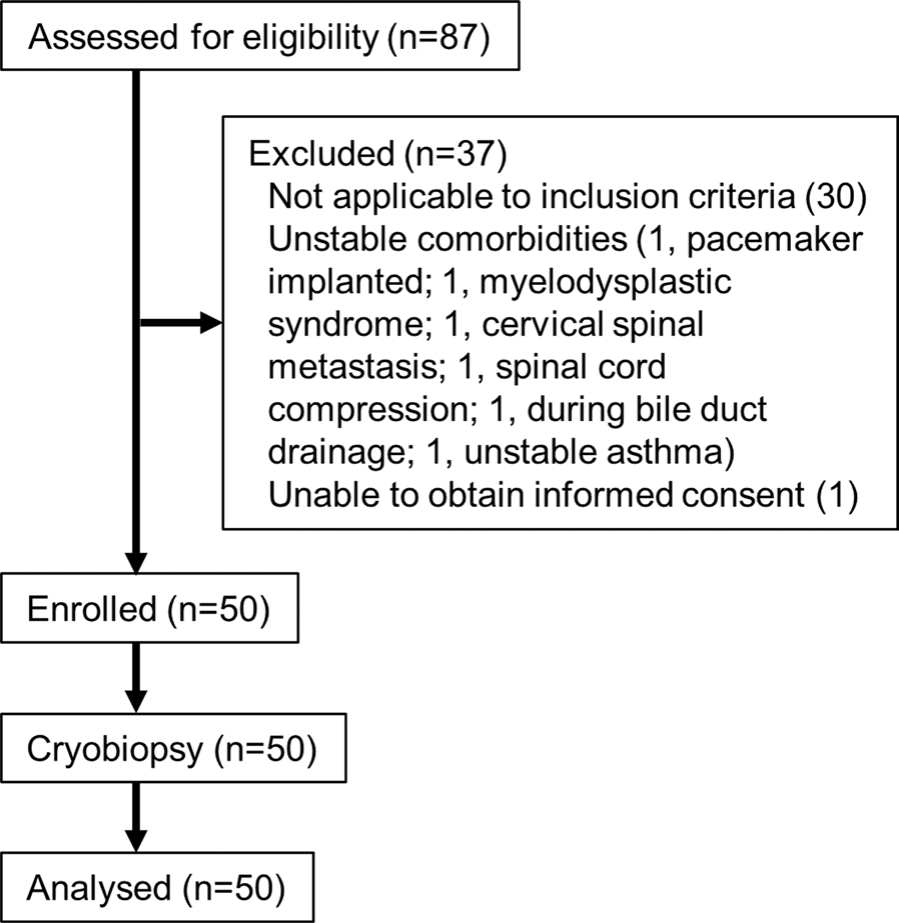
Flow diagram illustrating patient enrolment

In total, 87 patients with 88 lesions were scheduled to undergo bronchoscopy for PPLs at our institution between July 15 and October 8 2020. Of the 57 patients who met the eligibility criteria, 6 were excluded because of unstable comorbidities and 1 could not give consent. Therefore, we enrolled the remaining 50 patients (Fig. 1). The baseline characteristics are shown in Table 1.The median age of the patients was 70 years (range 41–87 years) and 70% were male. The median size of the target lesions was 20.8 mm (range 8.2–29.6 mm), and 44% were less than 20 mm. Most lesions were located in the right upper lobe/left upper segment (54%) and in the outer area (86%). The negative bronchus sign was seen in 17 patients (34%).

**Table 1:** Patient characteristics

Variable	*n* = 50
Age (years)	70 (41–87)
Sex	
Male	35 (70.0)
Female	15 (30.0)
Lesion size (mm)	20.8 (8.2–29.6)
≤ 20	22 (44.0)
> 20	28 (56.0)
Lobe	
Right upper lobe/left upper segment	27 (54.0)
Right middle lobe/left lingula	9 (18.0)
Right/left lower lobes	14 (28.0)
Location	
Outer	43 (86.0)
Inner	7 (14.0)
Morphology	
Solid	31 (62.0)
Part-solid	19 (38.0)
Distance from the costal pleura (mm)	9.8 (0.0–40.3)
≤ 10	26 (52.0)
> 10	24 (48.0)
Bronchus sign	
Positive	33 (66.0)
Negative	17 (34.0)
Related bronchial generation	
≤ 6	14 (28.0)
> 6	36 (72.0)
Visibility on plain radiograph	
Visible	38 (76.0)
Invisible	12 (24.0)

Data are presented as number (%) or median (range)

All 50 lesions could be detected using R-EBUS; the probe was located within and adjacent to the lesion in 26 (52%) and 24 (48%) cases, respectively. Thus, all patients underwent cryobiopsy and were included in the analysis (Fig. 1).

The overall diagnostic yield was 94% (47/50 patients; 95% CI, 83.5–98.8%). No differences in diagnostic yield were observed depending on the position of the R-EBUS probe; the yields within and adjacent to the lesion were 92.3% (24/26) and 95.8% (23/24), respectively. Factors affecting the diagnostic yield are shown in Table 2; the positive bronchus sign had a significantly higher diagnostic yield than did the negative bronchus sign (100% vs. 82.4%; *P* = 0.035).

**Table 2:** Factors affecting the diagnostic yield of cryobiopsy

Variable	Diagnostic case, n	Diagnostic yield, %	*P*-value
Age (years)			0.103
≤ 70	26/26	100.0	
> 70	21/24	87.5	
Sex			1.000
Male	33/35	94.3	
Female	14/15	93.3	
Lesion size (mm)			1.000
≤ 20	21/22	95.4	
> 20	26/28	92.9	
Lobe			0.704
Right upper lobe/left upper segment	25/27	92.6	
Right middle lobe/left lingula	9/9	100.0	
Right/left lower lobes	13/14	92.9	
Location			0.370
Outer	41/43	95.3	
Inner	6/7	85.7	
Morphology			1.000
Solid	29/31	93.5	
Part-solid	18/19	94.7	
Distance from the costal pleura (mm)			0.602
≤ 10	25/26	96.1	
> 10	22/24	91.7	
Bronchus sign			0.035
Positive	33/33	100.0	
Negative	14/17	82.3	
Related bronchial generation			1.000
≤ 6	13/14	92.9	
> 6	34/36	94.4	
Visibility on plain radiograph			1.000
Visible	36/38	94.7	
Invisible	11/12	91.7	
R-EBUS finding			1.000
Within	24/26	92.3	
Adjacent to	23/24	95.8	

*R-EBUS* radial endobronchial ultrasound

The final diagnoses are listed in Table 3. The most frequent histopathological diagnosis was adenocarcinoma. Of the 47 patients diagnosed using cryobiopsy, two were diagnosed by stamp cytology and not histology. The three patients who could not be diagnosed using cryobiopsy were confirmed by surgery to have adenocarcinoma in one and squamous cell carcinoma in the other two. Thus, the sensitivity, specificity, and accuracy of cryobiopsy for the diagnosis of a malignant nodule were 93.8%, 100%, and 94.0%, respectively. Moreover, the diagnostic yield of stamp cytology was 74.0% (37/50), whereas it was 68.0% (34/50) for liquid cytology.

**Table 3:** Final diagnosis

	Diagnostic case (*n* = 47)	Non-diagnostic case (*n* = 3)
Adenocarcinoma	31	1
Minimally invasive adenocarcinoma	1	0
Squamous cell carcinoma	5	2
Adenosquamous carcinoma	1	0
LCNEC	1	0
Metastatic tumour	4	0
Lymphoepithelioma-like carcinoma	1	0
Carcinoid tumour	1	0
Tuberculosis	1	0
Organizing pneumonia	1	0

*LCNEC* Large-cell neuroendocrine carcinoma

**Fig. 2:**
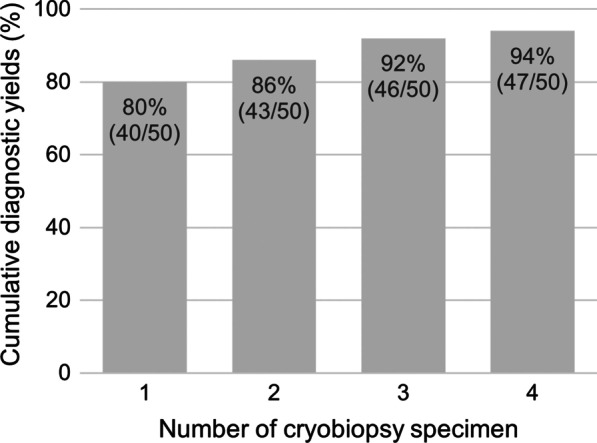
Cumulative diagnostic yield according to the number of cryobiopsy specimens. It improves as the number of specimens increases and seems to reach a plateau after three specimens

The median number of cryobiopsies performed per patient was four (range, one to five biopsies), and four specimens each were obtained from most of the patients (36/50, 72%). Of all the biopsies performed, a specimen was not obtained in one biopsy, and thus the patient underwent five biopsies to obtain four specimens. The major reason for discontinuing cryobiopsy was moderate bleeding. Taking only the first biopsy specimen into account, 80% (40/50) of patients were diagnosed. The cumulative yield was 86% (43/50) in the second biopsy specimen, 92% (46/50) in the third, and 94% (47/50) in the fourth (Fig. 2). Furthermore, among the 164 biopsies performed in 47 diagnostic cases, 54 (31.1%) biopsy specimens were non-diagnostic.

**Fig. 3:**
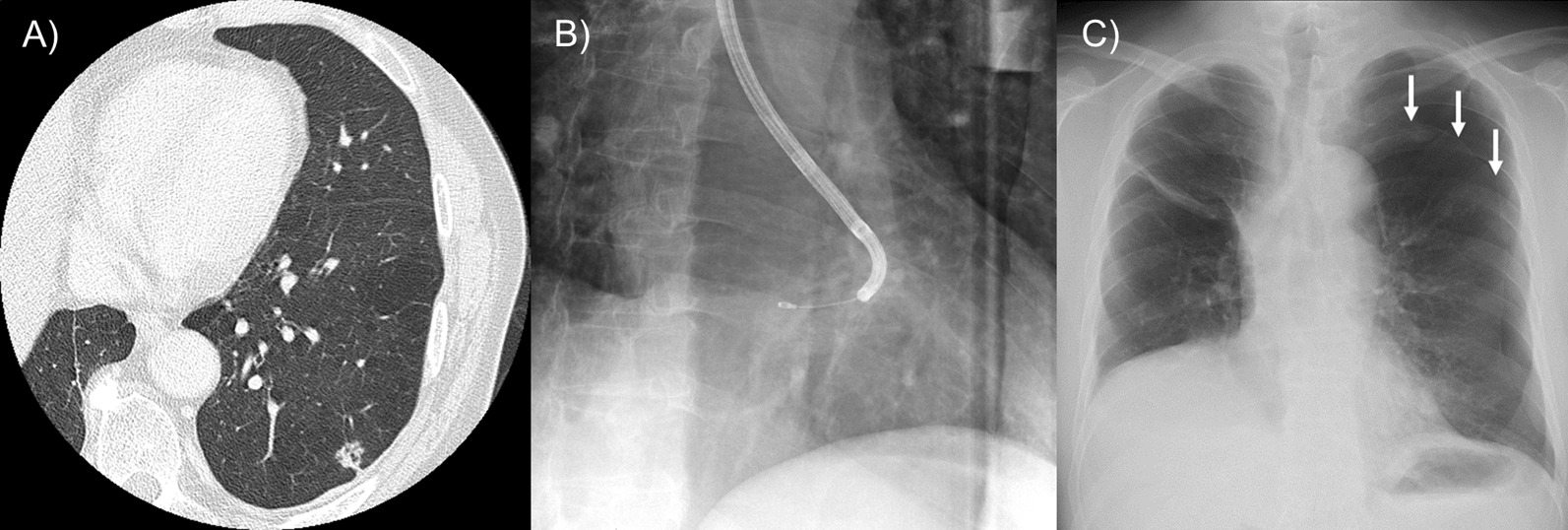
Representative case 1 of a patient who developed pneumothorax after cryobiopsy. **A** Computed tomography image showing a 12.4-mm solid nodule in the left lower lobe adjacent to the costal pleura. **B** The right anterior oblique view of the X-ray fluoroscopic image during cryobiopsy. The positional relationship between the pleura and cryoprobe could not be confirmed. **C** Chest plain radiograph acquired after the procedure revealed left pneumothorax (arrows)

The adverse events are summarised in Table 4. Mild and moderate bleeding were identified in 14 (28%) and 31 (62%) patients, respectively. Severe bleeding was observed in one patient (2%) who required no further intervention other than suction and transbronchial instillation of thrombogenic agents. Moderate pneumothorax necessitating chest drainage and pneumonia were observed in one patient each. A representative case of pneumothorax is shown in Fig. 3.

**Table 4:** Adverse events

	Number (%)
Bleeding	
None	4 (8.0)
Mild	14 (28.0)
Moderate	31 (62.0)
Severe	1 (2.0)
Life-threatening	0 (0.0)
Pneumothorax	1 (2.0)
Pneumonia	1 (2.0)

## Discussion

To our knowledge, this is the first prospective study to evaluate the diagnostic accuracy of the new 1.7-mm cryoprobe for PPLs. This study demonstrated the feasibility of transbronchial cryobiopsy using the new cryoprobe, suggesting its potential as a tool with excellent diagnostic accuracy. We achieved a diagnostic yield of 94% (95% CI, 83.5–98.8%), which exceeded the expected yield of 85%, and the lowest limit for 95% CI exceeded the threshold yield of 70% that was estimated on the basis of the findings of previous studies on conventional devices. Nevertheless, a certain percentage of patients had factors that were generally considered poorly diagnosed using bronchoscopy, such as small lesions less than 20 mm in diameter and a negative bronchus sign [4, 6, 7, 10, 12]. The new cryoprobe may also be effective in diagnosing lesions that are difficult to access or have a poor diagnostic yield with conventional devices.

Numerous studies reported that the diagnostic yield of forceps biopsy for PPLs was higher (73–84%) when the R-EBUS probe was within the lesion, as opposed to being adjacent to it (43–61%) [5]. This is possibly due to the design of the forceps that enables grasping a tissue only in the forward area, but not the lateral area. In contrast, our study showed that cryobiopsy wherein the R-EBUS probe was positioned adjacent to the lesions had comparable diagnostic yields to cryobiopsy wherein the R-EBUS probe was positioned within the lesions. Similar results were reported for conventional cryoprobes [20, 23]. These suggested that cryobiopsy could be useful even when the R-EBUS probe was adjacent to the lesions. This was attributed to the fact that the cryoprobe allowed biopsy sampling from lateral areas because the tissue surrounding the tip of the probe was frozen. This is another advantage of cryoprobes over forceps, in addition to obtaining better quality and quantity of tissues.

**Fig. 4:**
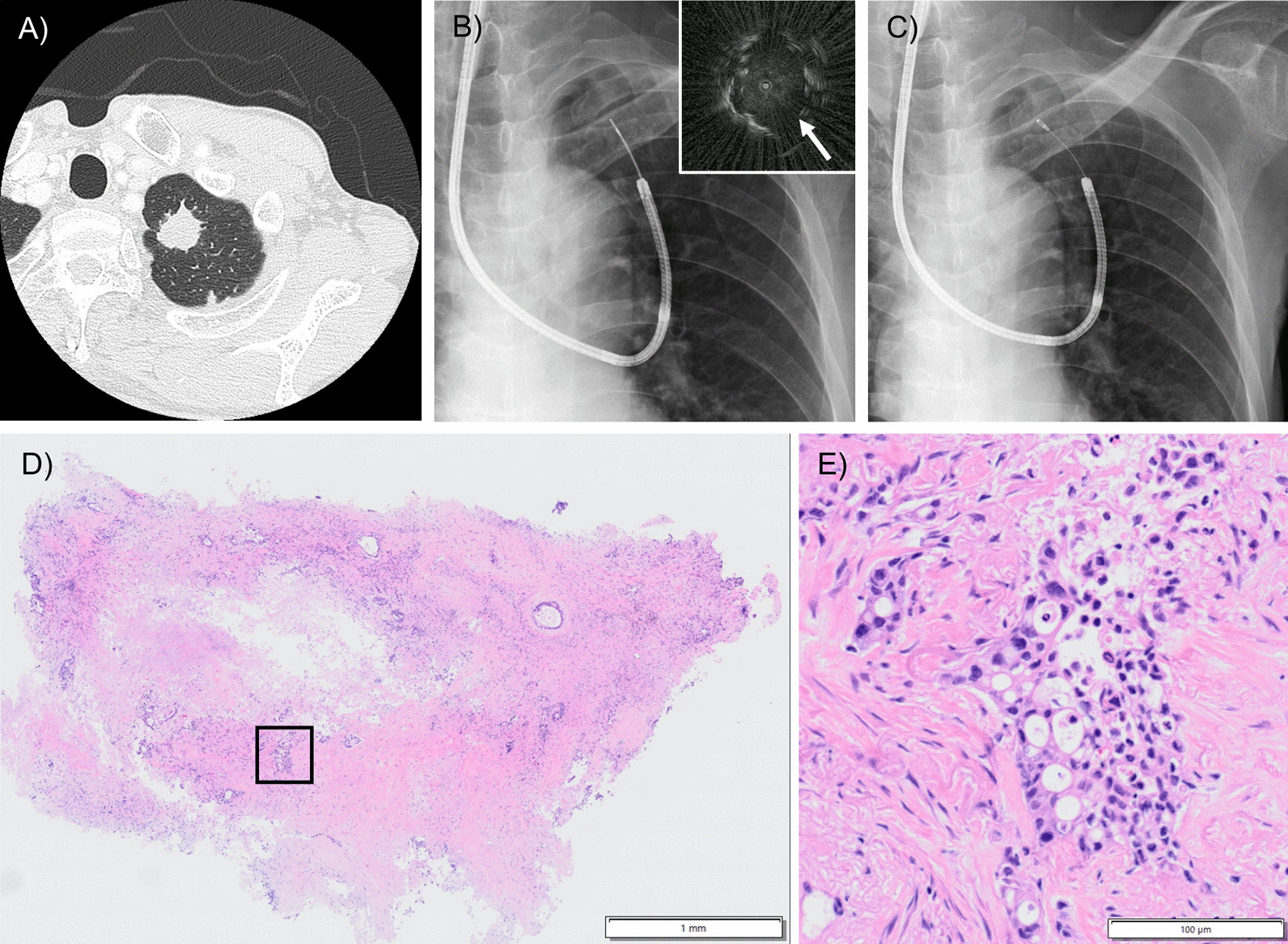
Representative case 2 with a target lesion in a previously difficult-to-reach area. **A** Computed tomography image revealing a pulmonary nodule 21.8 mm in diameter in the left upper segment 1 + 2. **B** The location of the lesion is confirmed by radial endobronchial ultrasound (R-EBUS), within the lesion (arrow), under X-ray fluoroscopic guidance. **C** X-ray fluoroscopic image showing the 1.7-mm cryoprobe reaching the lesion as smoothly as the R-EBUS probe because of its flexibility. **D** Hematoxylin and eosin staining of the specimen (magnification, × 10). **E** Higher magnification (square) revealing adenocarcinoma in which tumour cells proliferate while forming glandular structures (magnification, × 100). The stained tissue sections were scanned with 40 × objective using a high-resolution digital slide scanner (SLIDEVIEW VS200, Olympus, Tokyo, Japan) and viewed using VS200 ASW (version 3.1, Olympus). All images were scanned at 24-bit true colour and image manipulation was not performed

Although the difference between the number of diagnostic and non-diagnostic results was small, the presence of the bronchus sign was significantly associated with an increased diagnostic yield. This finding is consistent with those of previous studies showing that the CT bronchus sign is a strong predictor of successful diagnosis for every bronchoscopic technique [1, 5, 11, 22]. In terms of other factors, the diagnostic yield was not significantly affected by the following variables: lobe, location, distance from the costal pleura, and related bronchial generation. The conventional cryoprobe is thick and stiff and poses difficulties when approaching some locations, such as the subpleural areas and upper lobes. Moreover, these features may lead to inaccurate placement of the probe when the route to the lesion contains multiple branches. In contrast, the new thin and flexible cryoprobe could be extended to the distal bronchus, thus enabling easier and more appropriate placement of the probe at the same site confirmed using R-EBUS even in previously difficult-to-reach areas, as demonstrated in our representative case (Fig. 4).

Despite these advantages, using the new cryoprobe poses some challenges. The biopsy specimen still has to be removed together with the endoscope, as it cannot be retracted through the working channel as with the conventional cryoprobe. This necessitates re-navigation and re-insertion of the bronchoscope and cryoprobe even in cases of bleeding from the biopsy site, thereby raising the possibility that the cryoprobe cannot be consistently introduced to the correct location. This may explain why approximately one-third of the biopsy specimens were nondiagnostic in this study. Furthermore, we calculated the cumulative yield to be 80%, 86%, 92%, and 94% for the first, second, third, and fourth biopsy specimens, respectively. These results suggest that three biopsies are necessary to optimise the diagnostic value of the new cryoprobe.

Cytological examination, with a diagnostic yield for stamp and liquid cytology of 74.0% and 68.0%, respectively, was not very effective for detecting malignancy. Meanwhile, two lesions were identified as atypical based on tissue samples but were identified as malignancies on stamp cytology. Similar findings have been reported in a study using a conventional cryoprobe [21]. Owing to the large size of the tissue samples obtained using cryobiopsy, the sectioned area may not contain tumour cells if their distribution is uneven. Although little evidence exists regarding this discrepancy between histological and cytological findings, cytological findings may be important to increase the chances of a diagnosis.

Another major concern with cryobiopsy is the possibility of complications, mainly bleeding and pneumothorax. Although there was heterogeneity in the grading of bleeding severity and the method used to control bleeding, cryobiopsy reportedly induced more bleeding than did forceps biopsy; mild to moderate bleeding occurred in 47–72% of PPLs, whereas the rate of severe bleeding was 0.5% [18–23]. Similarly, the most frequently observed complication in this study was mild to moderate bleeding. Nevertheless, it could be controlled in all patients, including a case of severe bleeding, by using the two-scope method without further interventions [29]. Pneumothorax occurred in one patient (2%), and its incidence did not differ from that of forceps biopsy (0–5.1%) [1, 4]. However, the larger specimen obtained using the cryoprobe may have increased the rate of chest tube placement due to more damage to the pleura; for forceps biopsy, the reported rate was only 0.4%.

Our study has some limitations. First, this study was performed at a single institution with a relatively small sample size calculated on the basis of the width of the 95% CI rather than statistical power. Our institution is a high-volume centre that diagnoses approximately 600 PPLs by bronchoscopy each year, and the operators in this study were limited to three experts with at least 100 cases of experience performing cryobiopsy using conventional cryoprobes. Thus, our results may have limited generalizability. Nevertheless, the new cryoprobe clearly improves the reachability of PPLs, and the results shown, which were collected and presented as procedures performed according to a protocol to ensure an objective analysis immediately after its introduction, would be practical enough for institutions that routinely perform bronchoscopic diagnosis of PPLs. Second, this pilot study did not compare the new cryoprobe with other sampling devices. Further large-scale trials in a randomised setting are required to establish the true diagnostic value of cryoprobes.

## Conclusion

In conclusion, this study demonstrated the potential of the new 1.7-mm single-use cryoprobe as an effective tool with excellent diagnostic accuracy and tolerable safety for diagnostic bronchoscopy of PPLs.

## Data Availability

All data generated or analysed during this study are included in this article. Further enquiries can be directed to the corresponding author on reasonable request.

## Abbreviations

CI: Confidence interval
PPL: Peripheral pulmonary lesion
R-EBUS: Radial endobronchial ultrasound
